# Beyond Canonical CO Oxidation: Structural and Evolutionary Insights Into a Non‐Canonical Carbon Monoxide Dehydrogenase

**DOI:** 10.1002/anie.1702233

**Published:** 2026-07-24

**Authors:** Maximilian Böhm, Vivek Srinivas, Benjamin Wiseman, Ping Huang, Moritz Senger, Martin Högbom, Henrik Land

**Affiliations:** ^1^ Department of Chemistry – Ångström Laboratory Uppsala University Uppsala Sweden; ^2^ Department of Biochemistry and Biophysics and Science for Life Laboratory Stockholm University Stockholm Sweden; ^3^ Department of Chemistry for Life Sciences Uppsala University Uppsala Sweden

**Keywords:** bioinorganic chemistry, CODH, cryo‐EM, EPR spectroscopy, metalloproteins

## Abstract

Carbon monoxide dehydrogenases (CODHs) catalyse the reversible oxidation of CO to CO_2_ and play central roles in microbial carbon metabolism. While well‐characterised CODHs from different phylogenetic backgrounds exhibit high bidirectional activity, the enigmatic clade B remains functionally uncharacterised. Here, we present the first structural and biochemical characterisation of a clade B CODH from *Ruminococcus flavefaciens* (*Rf*CODH). It reveals striking divergence from canonical enzymes. A new anaerobic cryo‐EM workflow was developed, carried out entirely under anoxic conditions by manual blotting and plunge freezing. It resulted in a 2.53 Å *Rf*CODH structure. The structure adopts the typical CODH fold, but exhibits blocked gas channels, a compromised proton transfer pathway and disrupted cofactor coordination. This provides a structural rationale for *Rf*CODH's severely attenuated CO oxidation activity (13 mU/mg vs. 900 U/mg for the well‐studied *Ch*CODH‐II). EPR spectroscopy reveals unique oxidised C‐cluster states not previously characterised in CODHs. Mirror tree analysis hints to co‐evolution between clade B CODHs and associated ABC transporter substrate‐binding proteins, suggesting these enzymes function in metabolism of substrates imported via the ABC transporter module. All findings indicate evolutionary repurposing of the CODH scaffold for alternative physiological functions.

## Graphical abstract

Clade B carbon monoxide dehydrogenases (CODHs) remain enigmatic, diverging from canonical CODHs in structure and function. Here, we present the first characterization of *Ruminococcus flavefaciens* CODH (*Rf*CODH), revealing occluded substrate pathways and attenuated CO oxidation activity. Cryo‐EM, EPR spectroscopy and co‐evolution analysis with ABC transporters hint at alternative substrates and evolutionary repurposing. This study reshapes our understanding of CODH diversity and microbial carbon metabolism.

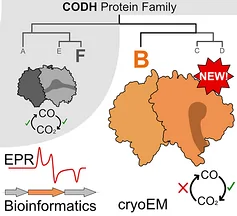

## 1 Introduction

Nickel‐iron carbon monoxide dehydrogenases (CODHs) catalyse the interconversion of CO and CO_2_, playing crucial roles in microbial carbon metabolism and energy conservation [1]. These enzymes are homodimeric and carry five metal clusters: two buried [4Fe4S] B clusters and one [4Fe4S] D cluster on the interface of the homodimer; substrate conversion takes place at the two biologically unique [Ni3Fe4S] C clusters with an additional Fe coordinated in proximity. These clusters are buried deep within the protein [1, 2]. Substrate access is regulated by proton transfer pathways mainly consisting of charged histidine residues [3], and hydrophobic gas channels [4].

Much of the biochemical diversity within the CODH family remains largely unexplored. While substantial progress has been made in understanding important features such as structure, catalytic mechanism and oxygen sensitivity, these studies only used CODH from phylogenetic clades A [5], E [6] and F [7, 8]. Meanwhile, clades C [9] and D [10] have only one biochemically characterised representative each, and clades B, G, and H lack any characterisation despite their widespread distribution in microbial genomes [11, 12]. Particularly eye‐opening was the study of the first clade D CODH from *Carboxydothermus hydrogenoformans*, *Ch*CODH‐V [10]. It showed an altered nickel‐free C cluster and no CO_2_ reduction, nor CO oxidation activity [10]. Only hydroxylamine could be identified as a substrate [10], suggesting clade D CODHs possess a different role in the organism than canonical CODHs. This supported the claims made by bioinformatic studies, that different clades serve different functions [12, 13] and hence perform different chemical reactions.

Clade B CODHs are particularly enigmatic for a different reason. First, they are primarily found in symbiotic host‐associated environments [11]. Secondly their genomic context shows striking differences from canonical CODHs: the majority of clade B CODHs are encoded within operons containing complete ABC transporter systems, comprising transmembrane domains (TMs), nucleotide‐binding domains (NBDs), and substrate‐binding proteins (SBPs) [12]. Additionally, many clade B operons contain two conserved hypothetical proteins whose functions remain unknown but appear across diverse organisms including bacteria from the phyla *Bacillota* and *Pseudomonadota*.

The presence of a CODH in *Ruminococcus flavefaciens* was noted as puzzling as early as 2004, when Antonopoulos and colleagues observed its gene [14]. Even though it was known that *R. flavefaciens* shows increased growth on high carbonate [15], the presence of a CODH gene does not align with what is known about the overall metabolism of this gut microbe. This observation hints at a broader question: do clade B CODHs actually function as carbon monoxide dehydrogenases, or has this enzyme scaffold been repurposed for alternative chemistry? Much like the clade D CODH, *Ch*CODH‐V, with an until now unknown function and an altered C cluster [10].

Here, we present the first biochemical and structural characterisation of a clade B CODH from *Ruminococcus flavefaciens* (*Rf*CODH). It is encoded in an operon encompassing the canonical clade B architecture: the CODH gene flanked by ABC transporter components and two conserved hypothetical proteins. Through a combination of solution‐based activity assays, electron paramagnetic resonance (EPR) and FTIR spectroscopy, cryo‐electron microscopy (cryo‐EM) and phylogenetic co‐evolution analysis, we demonstrate that *Rf*CODH exhibits severely attenuated CO oxidation activity, which is several orders of magnitude lower than characterised clade F enzymes. *Rf*CODH furthermore lacks detectable CO_2_ reduction capability above the detection limit of our solution‐based assay. For structure determination, we developed a new anaerobic grid preparation workflow using simple cross‐locking tweezers for manual blotting and manual plunge freezing into liquid ethane, with grids stored submerged in the ethane cup, avoiding any introduction of liquid nitrogen into the glovebox. The 2.53 Å cryo‐EM structure reveals that while *Rf*CODH maintains the overall CODH fold and accessory metal cluster architecture, its substrate access pathways are systematically occluded by bulky hydrophobic residues, and its proton transfer pathway is altered. We were also not able to fully resolve the active site C‐cluster, likely due to disorder or alternate states, but the nature of the coordinating residues suggests a non‐canonical conformation. Bioinformatic mirror tree analysis hints towards co‐evolution between clade B CODHs and their associated SBPs, suggesting functional coupling. Together, these findings indicate that clade B CODHs represent a functionally divergent lineage that has retained the versatile CODH/hybrid cluster protein scaffold while evolving toward alternative physiological roles.

## 2 Results and Discussion

### 2.1 Activity of *Rf*CODH

**FIGURE 1:**
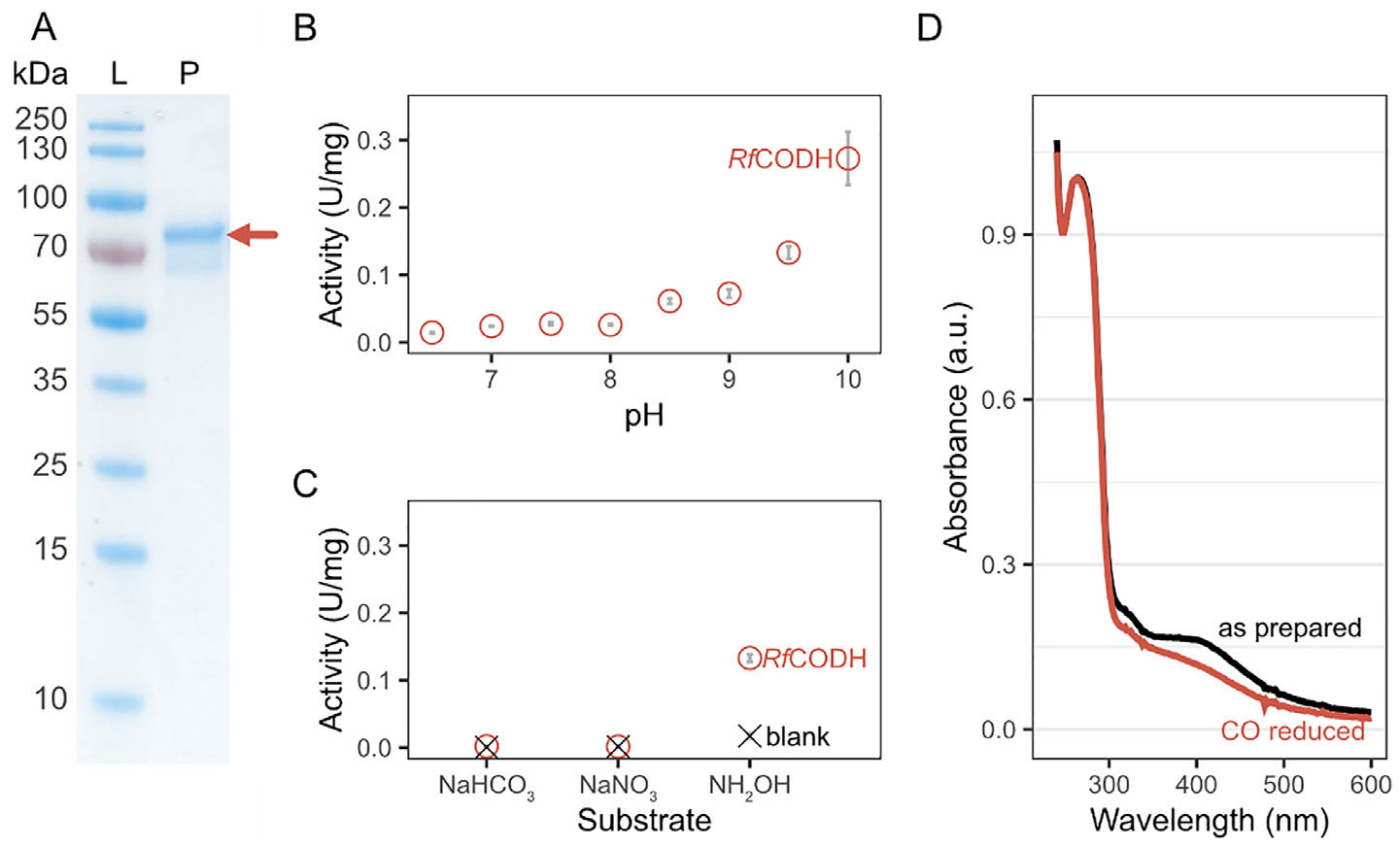
Biochemical characterisation of *Rf*CODH. (A) SDS‐PAGE analysis of purified *Rf*CODH (P) showing a clear band for *Rf*CODH at 79 kDa with PageRuler Plus Protein Ladder (L, ThermoFisher 26619). For uncut gel see Figure S1. (B) pH dependency of *Rf*CODH CO oxidation activity. One unit (U) is defined as the reduction of one µmol of methyl viologen (MV) per minute. All measurements were performed as technical triplicates. Error bars reflect the standard error. (C) Auxiliary activities of *Rf*CODH towards NaNO_3_, NaHCO_3_, and NH_2_OH, measured in 50 mM TAPS‐NaOH, 20 mM NaCl, pH 8. One U is defined as one µmol of MV oxidised per minute. All measurements were performed as technical triplicates. Error bars reflect the standard error. (D) UV–vis spectra of *Rf*CODH with buffer (50 mM Tris‐HCl, 20 mM NaCl, pH 8) (black) and CO‐saturated buffer (red).

*Rf*CODH, tagged with a C‐terminal Strep‐tag II, expresses well and is readily purified from *Escherichia coli* cell lysate. Figure 1A shows an SDS‐PAGE analysis with a clear band at the expected molecular weight of 79 kDa for *Rf*CODH. The metal content after purification was 6–8 Fe/monomer and 0.2–0.5 Ni/monomer, determined via ICP‐OES and TXRF; for full metalation of a canonical CODH we expect 10 Fe/monomer and 1 Ni/monomer. The Fe‐ and Ni‐content is assumed to be slightly underestimated due to the presence of small amounts of impurities (Figures 1A and S1). The protein exhibits a low activity towards CO oxidation, but only after preincubation in CO saturated buffer. CO oxidation was tested over a range of pH‐values (Figure 1B) and *Rf*CODH's activity increases with higher pH, which has been observed for other CODHs as well [16]. When *Rf*CODH is incubated with CO alone we observed a decrease in absorbance between 350 and 400 nm (Figure 1D), which is commonly seen in the reduction of [FeS] cluster proteins, potentially suggesting a reductive activation of the enzyme. However, preincubation with sodium dithionite (NaDT) did not activate the enzyme. We could not observe any CO_2_ reduction activity under standard CO evolution assay conditions, using methyl viologen and NaDT as electron mediator and donor, respectively [6]. Substituting electron mediator and donor to dmDQ‐H (3,11‐dimethyl‐7,8‐dihydro‐6*H*‐dipyrido[1,2‐*a*:2′,1′‐*c*][1,4]diazepine‐5,9‐diium) and europium EGTA (ethylene glycol‐bis(β‐aminoethyl ether)‐N,N,N′,N′‐tetraacetic acid) to increase overall driving force [17], also did not result in any observable activity. Auxiliary activities towards nitrate and hydroxylamine (NH_2_OH) were also tested using a methyl viologen oxidation assay. Only NH_2_OH seems to be a substrate for *Rf*CODH as it yielded significant methyl viologen oxidation (Figure 1C). We tested for CO_2_ and carbonate reduction that does not yield CO in the process, with no observable activity (Figure 1C). Compared to other CODHs, CO oxidation activity is three to five orders of magnitude lower (Table 1). One reason could be that *Rf*CODH was not fully matured in the expression host, which would explain the low Ni/monomer ratio. However, an alternative reason could be that CO is not the primary substrate for clade B CODHs. The NH_2_OH reduction activities are higher compared to the prototypical clade F CODH, *Ch*CODH‐II, at the same temperature, but low when compared to the reported values of prototypical hybrid cluster protein from *Pyrococcus furiosus* (*Pf*HCP) and clade F and D CODH, *Ch*CODH‐II and *Ch*CODH‐V [10, 18, 19, 20, 21]. Even though the reported values reflect the activity at elevated temperatures, this makes NH_2_OH a rather questionable substrate. Therefore, the optimum conditions for this enzyme, and its primary substrate are yet to be identified.

**TABLE 1:** Activity of CODH towards selected substrates. One U is defined as the conversion of one µmol of substrate per minute at pH 8, room temperature, and MV as electron shuttle.

		Activity in U/mg			
Name	Clade	CO oxidation	CO_2_ reduction	NH_2_OH reduction	Ref.
*Rf*CODH^a^	B	0.013 ± 0.007	n.a.	0.066 ± 0.003	This study
*Tk*CODH‐II^b^	C	13.9 ± 4.1	n.r.	n.r.	[9]
*Ch*CODH‐II^a^	F	900 ± 28.2	16.9 ± n.r.	0.7^c^	[18, 19, 21]
		280 ± 75.9	1.18 ± 0.026	0.009 ± 0.002	This study
*Ch*CODH‐IV^a^	F	85 ± 1.2	n.r.	n.r.	[19]
*Ch*CODH‐V^a^, ^c^	D	n.a.	n.a.	0.11 ± 0.01	[10]
*Pf*HCP^d^		n.r.	n.r.	4.5 ± 0.2	[20]

Not reported (n.r.), not active (n.a.)

^a^ Recombinantly produced protein;

^b^ activity measured at 66°C;

^c^ measured at 40°C;

^d^ activity measured at 70°C and pH 9.

### 2.2 Electron Paramagnetic Resonance Spectroscopy Reports on Functional *Rf*CODH Cofactors

**FIGURE 2:**
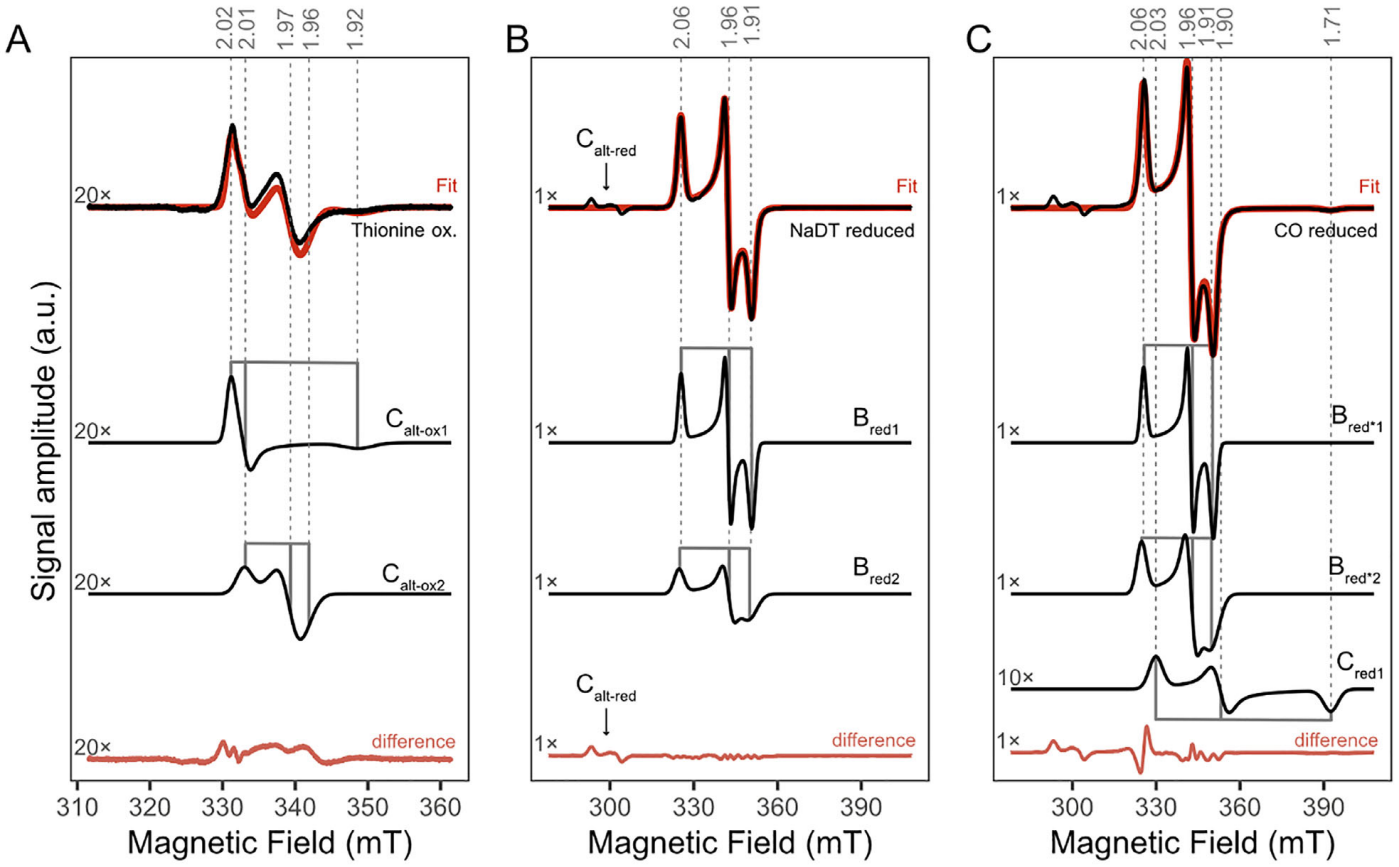
EPR spectra and simulations of *Rf*CODH. Experimental and simulated EPR spectra for (A) Thionine oxidised *Rf*CODH. (B) NaDT reduced *Rf*CODH. (C) CO reduced *Rf*CODH. Measured at 15 K, 15 G, 100 kHz, 9.37 GHz, and 2.5 mW.

Electron paramagnetic resonance (EPR) spectroscopy was employed to study the electronic state of *Rf*CODH's metal clusters. *Rf*CODH was incubated under different redox conditions to yield the following EPR spectra (Figures 2 and S2). Samples reduced with dithiothreitol (DTT), incubated with carbonate buffer or buffer with no additional substrates/redox substances were in a reduced state and show no differences in redox states of the clusters (compare spectra “As isolated” and “DTT reduced”, Figure S2). From this we conclude that our reductive purification procedure keeps *Rf*CODH in a reduced state, that cannot be further enriched with prolonged DTT exposure. Furthermore, carbonate (and hence CO_2_) was not able to oxidise the enzyme (compare spectra “As isolated” and “CO_2_ exposed”, Figure S2), which is in line with the lack of activity towards CO_2_ reduction we demonstrated above. All EPR signals from various species exhibit rhombic character. EPR‐active redox states are referred to by their associated metal cluster.

When *Rf*CODH is oxidised with thionine, we detected a spectrum which has not previously been characterised for CODH (Figure 2A and “Thionine oxidised” in Figure S2). Simulation suggests that this spectrum comprises two rhombic components that most likely stem from an altered oxidised C cluster and are therefore annotated as C_alt‐ox_ (C_alt‐ox1_, g_av_ = 1.984 [2.0216, 2.0105, 1.9187] and C_alt‐ox2_, g_av_ = 1.983 [2.0107, 1.9743, 1.9626]). Svetlitchnyi et al. (2001) observed a similar feature for *Ch*CODH‐I and *Ch*CODH‐II after exposure to oxygen [8]. But, the resolution of their spectra is too low to make meaningful comparisons. Interestingly, even though we determined a lower iron content than would be expected for a canonical CODH, no obvious indication of damaged FeS clusters could be observed in EPR, e.g., [3Fe4S] from degraded [4Fe4S]. The B and D clusters are in an oxidised EPR‐silent state under these conditions and do not contribute to the observed spectra.

However, when *Rf*CODH is reduced with NaDT, EPR spectroscopy shows features of two overlapping signals in the g 2.0 region that match with previously described signals from the B cluster (B_red1_, g_av_ = 1.974 [2.0573, 1.9561, 1.9080] and B_red2_, g_av_ = 1.976 [2.0617, 1.9543, 1.9113]) [8, 22, 23, 24, 25, 26, 27] (Figures 2B and S2). A signal with g_av_ = 2.232 [2.282, 2.213, 2.200], typical for d^9^ Ni^+^, appears along with other signals from B or C‐clusters (Figure 2B “difference”). This signal closely resembles the one previously reported by Basak et al [22]., though the signal reported here shows less rhombicity. It probably stems from a smaller population of Ni^+^ from an independently bound Ni^+^ or an altered reduced C‐cluster. We therefore denote it as C_alt‐red_. Even though it is likely that a Ni^+^ gives rise to this g_av_ ∼ 2.2 signal, we could not find a convincing fit, assuming a single spin ±1/2 transition. To further investigate this issue, the signal amplitudes (i.e., dχ″/dB, as an indicator of its susceptibility, χ), were measured under varied temperature and microwave power to yield Curie plots of two variations (Figure S3C, χ ∼ 1/T and S3D, χT ∼ T). The non‐linear correlation in both plots demonstrates spin transitions from a non‐“ground state only” behaviour even at temperature as low as 5 K. This result reinforces the assignment of an alternative C‐cluster—C_alt_.

After incubation with CO, *Rf*CODH's EPR signal changes to also include a new feature in the lower g ∼ 1.7 region (Figures 2C and S2) and additional intensity in the g ∼ 2.0 region. Simulation indicates that the signal is composed of three species, denoted as C_red1_, B_red1_ and B_red2_. The C_red1_ spectrum matches closely with the previously described reduced C cluster with a coordinated CO (C_red1_, g_av_ = 1.877 [2.0300, 1.8970, 1.7054]) [22, 25, 26, 27]. Power temperature studies on this signal can be seen in Figure S4. Simulations also show that the B_red_ signals shift slightly which is indicated with an * (B_red*1_, g_av_ = 1.973 [2.0551, 1.9556, 1.9091] and B_red*2,_ g_av_ = 1.977 [2.0612, 1.9553, 1.9148]), which can be rationalised as interaction with the now reduced C cluster. The g‐values for all reported states are summarised in Table S1.

### 2.3 Cyanide Probing Using IR Spectroscopy

**FIGURE 3:**
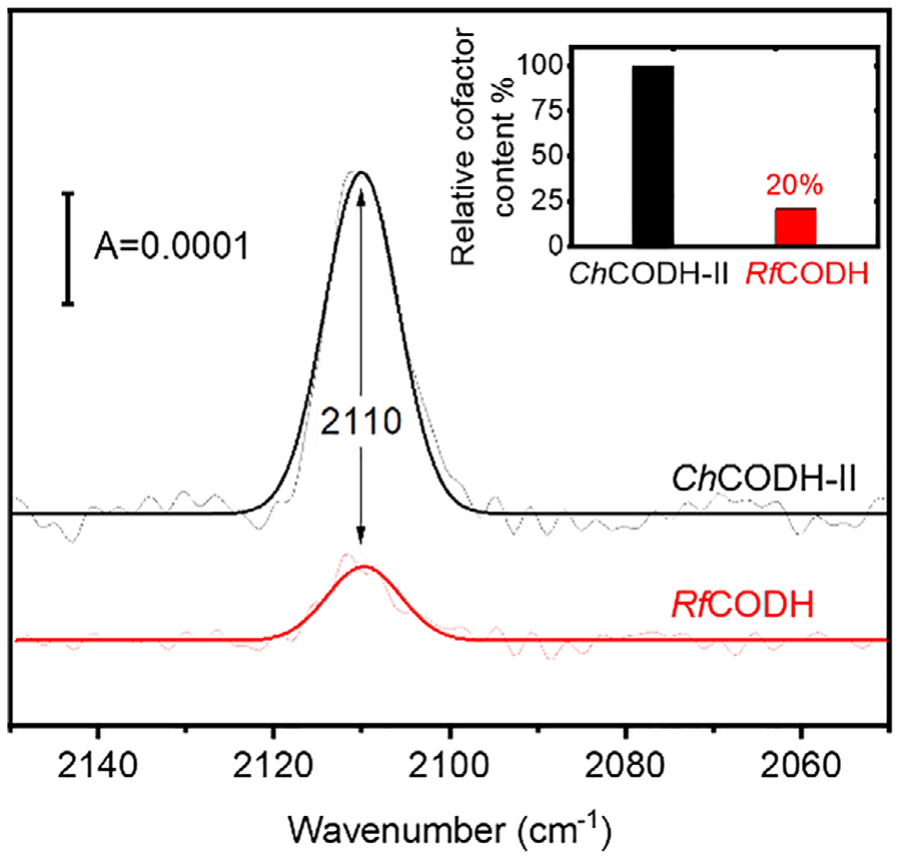
FTIR spectra of CN^−^‐bound CODHs. *Rf*CODH (red, below) and *Ch*CODH‐II (black, above) were incubated with CN^−^ following Ciaccafava et al. [28]. Enzymes were treated with potassium cyanide in Tris‐HCl buffer containing NaCl and NaDT, concentrated, and measured under anoxic conditions. Both enzymes show the characteristic vibrational band at 2110 cm^−1^ indicating CN^−^ bound to C clusters [28]. The absorbance signals were normalized using the amide II band as an indicator for enzyme concentration. Bold traces show fits, thin traces corrected data. Inset: Estimated relative cofactor concentration for *Rf*CODH (red) and *Ch*CODH‐II (black) assuming 100% cofactor integration for *Ch*CODH‐II.

To better understand the nature of the C cluster in *Rf*CODH and to compare it to canonical CODHs, such as *Ch*CODH‐II, we used cyanide (CN^−^) as an infrared (IR) sensitive probe. This has previously been shown to strongly inhibit the C‐cluster by binding to the nickel, leading to a distinct signal that can be used to probe the presence of a canonical C‐cluster [28]. We incubated each, *Rf*CODH and *Ch*CODH‐II, with potassium cyanide to generate cyanide bound forms following an established protocol [28]. The resulting IR spectra in the CN^−^ wavenumber region can be seen in Figure 3. *Ch*CODH‐II showed an absorption at 2110 cm^−1^ as described previously for a C cluster with CN^−^ ligand bound to the nickel [28]. An identical, but less intense signal emerged after *Rf*CODH was treated with potassium cyanide even though the enzyme concentration was approximately double (Figure 3). The lower intensity can have different reasons. First, that only a fraction of *Rf*CODH has a typical C cluster. Secondly, that only a limited amount of CN^−^ reaches the active site. Thirdly, that the extinction coefficient for this C≡N vibration is lower in *Rf*CODH due to differences in the active site coordination sphere. Lastly, our *Rf*CODH preparation only incorporated 0.2–0.5 Ni/monomer, providing limited sites for cyanide to bind, which in turn leads to a weaker signal. If incomplete maturation is assumed to be the only limiting factor, analysis of signal intensity results in an estimated 20% C‐cluster incorporation compared to our *Ch*CODH‐II sample. Overall, we can conclude that some *Rf*CODH in our sample likely contains a classical C cluster, containing a nickel with an open coordination site accessible to cyanide as indicated by the identical cyanide band position when compared to *Ch*CODH‐II.

### 2.4 Structural Analysis of *Rf*CODH

**FIGURE 4:**
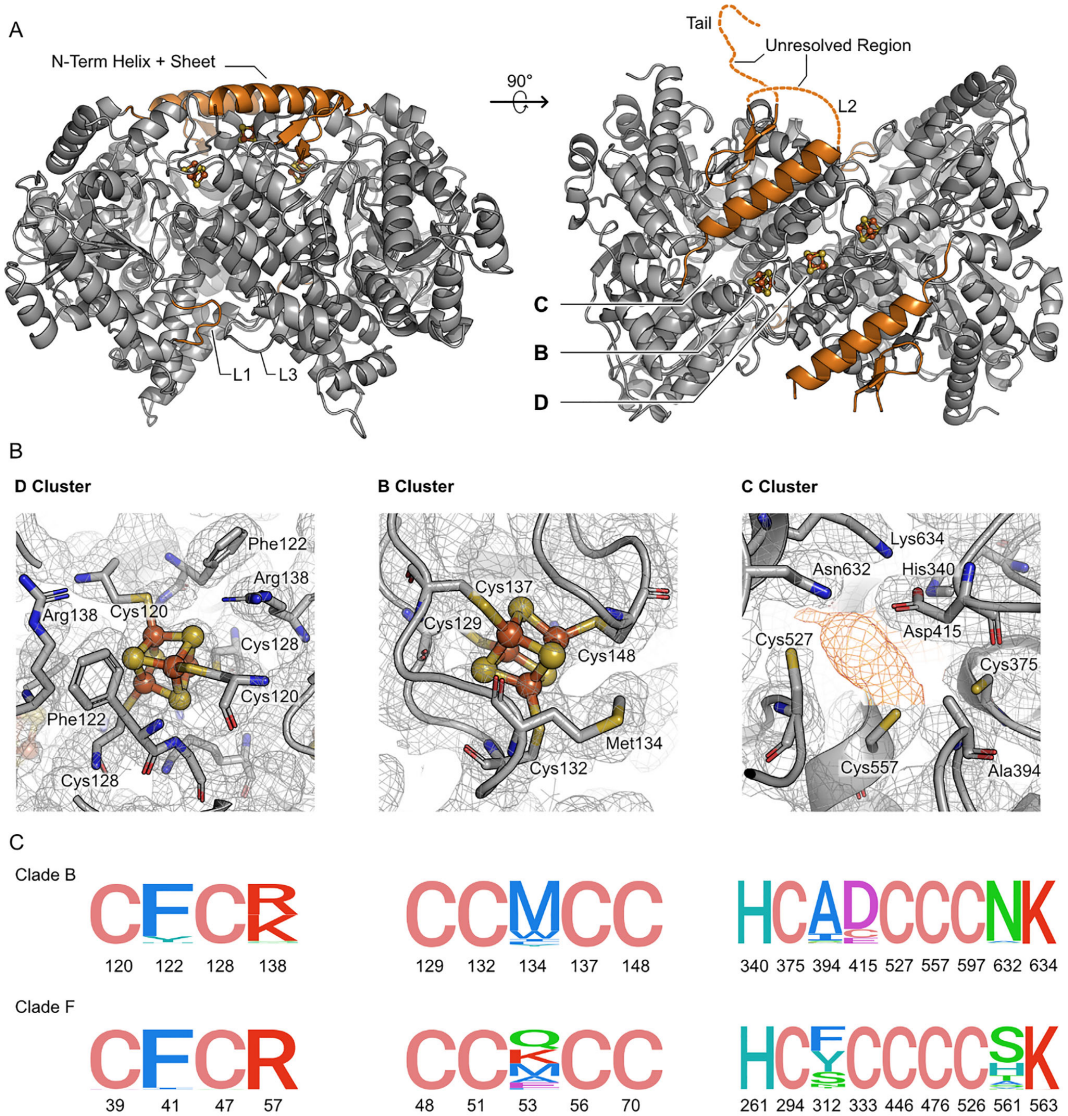
Structural overview of cryo‐EM structure of *Rf*CODH. (A) Top and side view of *Rf*CODH, with non‐canonical structural elements marked in orange, compared to clade F CODHs. (B) D, B and C cluster regions in *Rf*CODH. (C) Sequence logos of selected residues from cluster regions of clade B and clade F CODH, with *Rf*CODH and *Ch*CODH‐II numbering, respectively. The resolution distribution of the final *Rf*CODH volume can be seen in Figure S8. More detailed predicted volumes of ligand binding sites and chain differences are presented in Figure S9. DeepEMhancer [36] processed map was used for figures, a non‐uniform refinement map was used for model building.

To study the structural basis for *Rf*CODH's unusual catalytic properties, we determined its structure using cryo‐electron microscopy to an overall resolution of 2.53 Å (Figure 4). Sample application and vitrification of the grids was performed in a glovebox. We developed an anaerobic grid preparation workflow using solidified ethane to manage transfers through the glovebox antechamber and a deep well to maintain ethane at cryogenic temperature. This enables preparation of grids suitable for cryo‐EM studies of oxygen sensitive metalloproteins requiring minimum modifications to existing anaerobic gloveboxes. This method does not require dedicated plunge freezing robots inside anaerobic chambers [29, 30] or construction of custom anoxic chambers around such robots [31]. It maintains strict anaerobicity by transferring only solidified ethane into the glovebox while keeping liquid nitrogen external. This approach simultaneously reduces the risk of any gaseous expansion of liquid nitrogen inside the enclosure and removes the risk of oxygen contamination from foam dewars. Also, in contrast to blot‐free or chemically protected workflows [32], this protocol does not rely on oxygen scavengers such as dithionite to preserve metalloprotein integrity, thus avoiding unintended chemical reduction of other oxidised cofactor sites. The main limitation of this setup is manual blotting, which reduces reproducibility and may require additional grid screening compared to the protocols discussed above. The detailed setup (Figure S5) and workflow description are presented in the Supporting Information. Data collection and refinement workflow, as well as statistics are summarised in Figure S6 and Table S2.

*Rf*CODH shows a typical CODH fold and homodimeric structure (RMSD of 1.209 compared to *Ch*CODH‐II, PDB ID: 4UDX) with well‐resolved D and B clusters. Notably, *Rf*CODH contains an additional N‐terminal extension consisting of a histidine rich 35 amino acid long region (not resolved in the structure, due to its unordered nature), a small beta sheet, and an alpha helix (orange in Figure 4A). The helix is connected to the sheet via a structurally unresolved loop of 12 amino acids (L2). These elements are not present in other CODHs, though the functional significance of them remains unclear. An additional loop (L1) could be identified, not present in other characterised CODHs, which will be discussed in more detail later. The current structural resolution within the active site was not resolved enough to model a C cluster confidently (Figure 4B). This could either be due to a damaged protein during preparation, sample heterogeneity or due to alternate conformations of the C cluster. Figure 4C shows the sequence logo of cluster coordinating residues for clades B and F to compare sequence conservation between clades. As can be seen, C cluster coordination differs mainly at position 415, where in canonical clade F CODHs a thiol cysteine coordinates the [Ni3Fe4S] cluster (Cys333 in *Ch*CODH‐II). In almost all clade B enzymes, this is exchanged to a carboxylic acid residue (glutamate or aspartate, Asp415 in *Rf*CODH, Figures 4C and S7. FeS clusters are typically coordinated by cysteine thiolates but there are exceptions such as histidine and glutamate/aspartate [33]. Carboxylate coordination in many cases lead to a more labile and/or transient coordination as can be seen in e.g. the NO‐sensing NsrR protein, the [FeFe]‐hydrogenase maturation protein HydF and the previously mentioned *Ch*CODH‐V [10, 34, 35]. B and D cluster coordination is conserved between the two clades.

**FIGURE 5:**
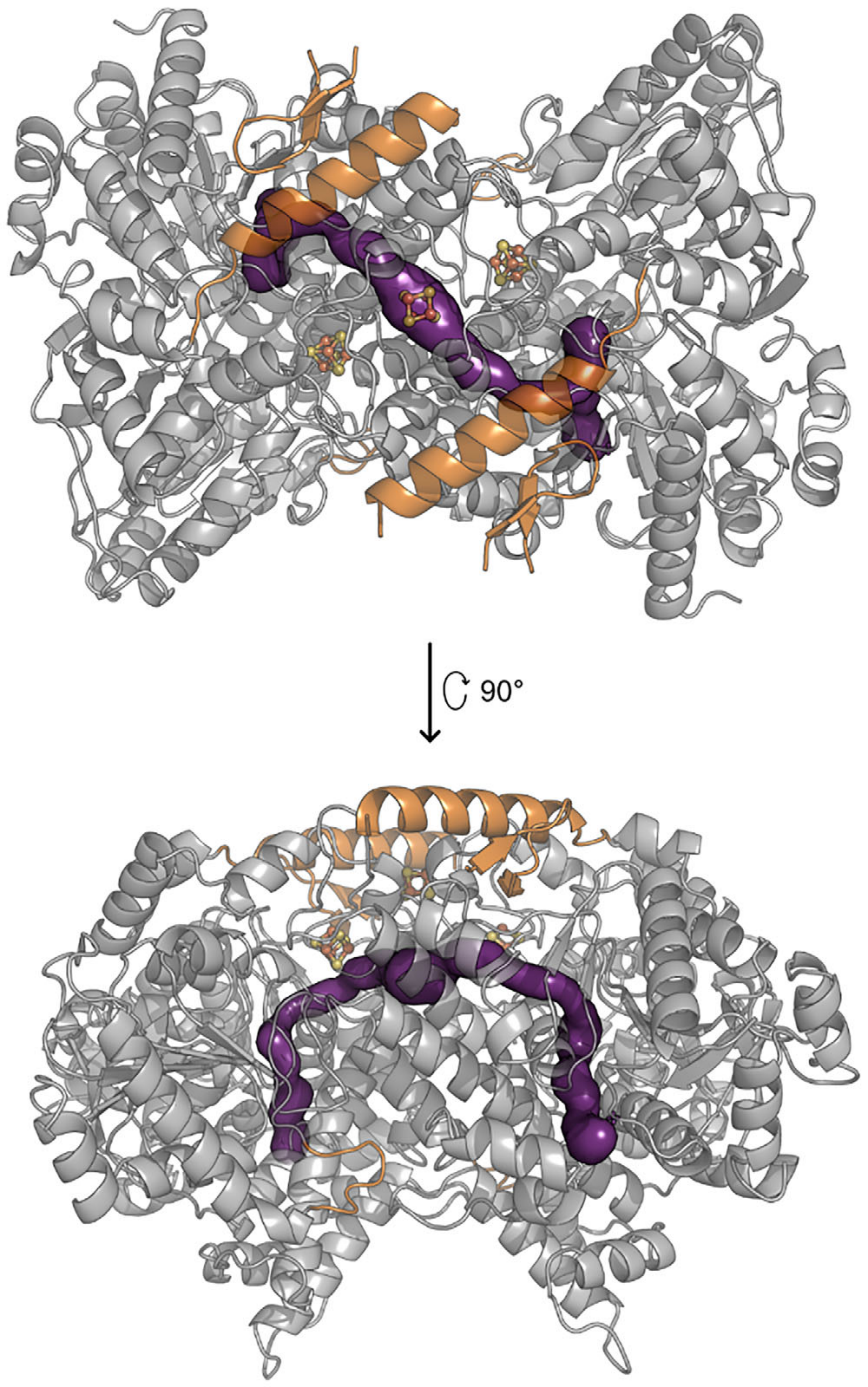
Gas channel of *Rf*CODH. The gas channel of *Rf*CODH (purple) was predicted with CAVER 3.0 [37] with a probe radius of 1.1 Å.

Despite the overall tertiary and quaternary structural conservation, closer examination reveals critical differences in substrate access pathways that rationalise *Rf*CODH's severely attenuated CO oxidation activity. We analysed the potential gas channels with CAVER 3.0 [37] and two different probe radii of 1.1 Å and 0.9 Å. We used Biester et al.’s structure from *Nitratidesulfovibrio vulgaris* CODH [4] *(Nv*CODH, formerly known as *Dv*CODH) to determine a probe radius that could replicate the gas channels observed in their structure based on xenon‐soaked crystals (PDB ID: 7TSJ). We found that a probe radius of 1.1 Å replicates the data, which we used for the primary analysis (Figure 5). We also analysed *Rf*CODH with the commonly used 0.9 Å probe radius (Figure S10). The results demonstrate that all but one of the canonical CO entry routes identified in *Nv*CODH are blocked in *Rf*CODH by bulky hydrophobic residues (see Figure S10, S11). The one non‐blocked canonical entry point is slightly shifted and smaller compared to *Nv*CODH, due to steric hindrance by Phe666 (Figure S10). We identified an alternative tunnel in *Rf*CODH leading directly to the presumed C‐cluster active site (Figure 5), which has not been seen in other CODHs before. This tunnel also connects to a cavity beneath the D‐cluster, similar to what was predicted by others for different CODHs using CAVER [4]. The architectural rearrangement of the gas channel suggests that if *Rf*CODH catalyses CO oxidation, substrate access must occur through a significantly more restricted pathway than in canonical CODHs, which usually have multiple entry and exit points. This could partly explain the lack of measurable activity against larger substrates such as CO_2_ and nitrate (Figure 1C), even though CO_2_ evidently is able to leave the active site. *Rf*CODH has some small gas channels predicted with small probe radius (see Figure S10), however, it contains only one bigger entry and exit point for substrate access to the active site (Figure 5).

**FIGURE 6:**
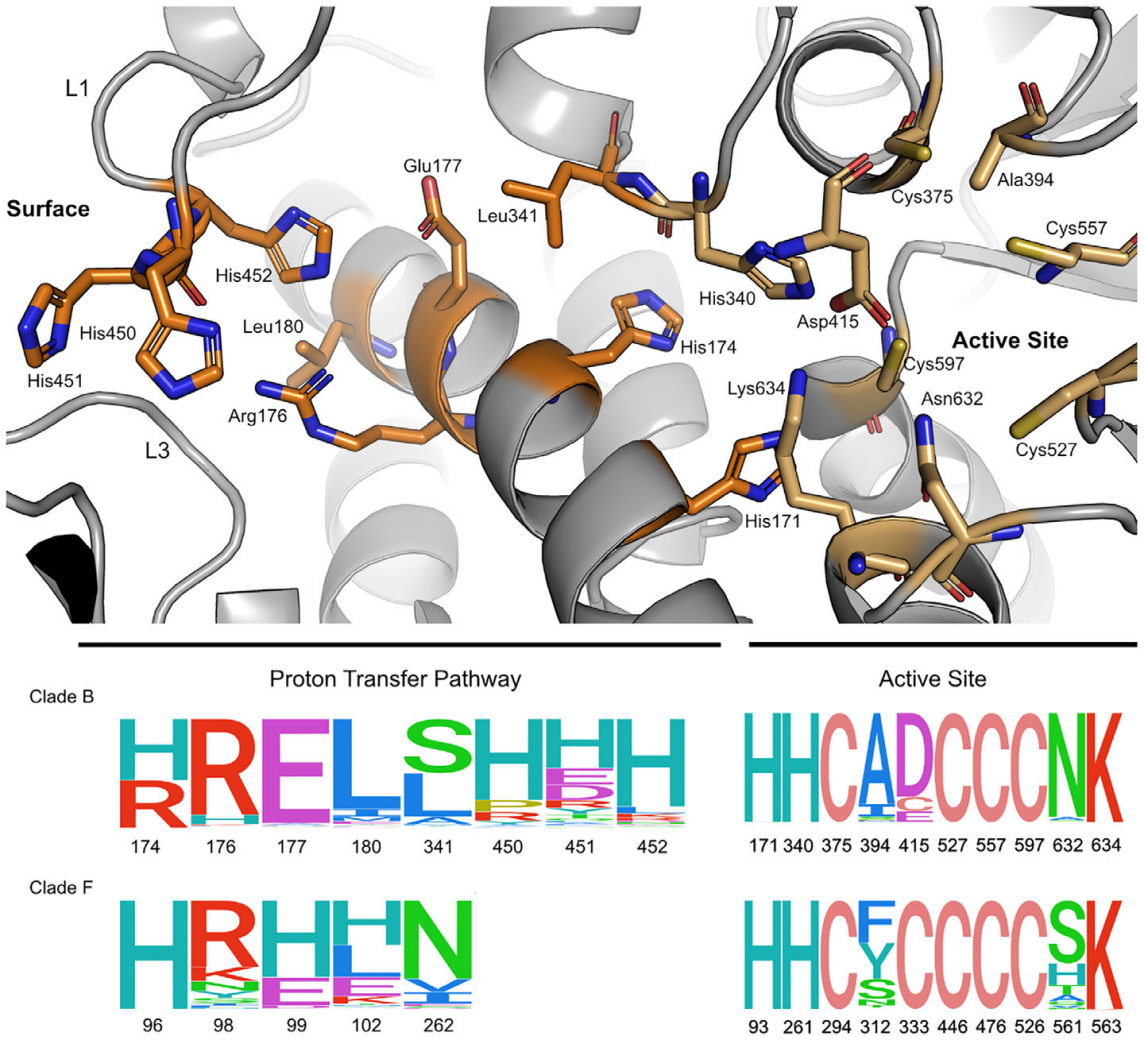
Putative proton transfer pathway. Top panel. Structure of putative proton transfer pathway (orange residues) and active site (gold residues) in *Rf*CODH (with *Rf*CODH numbering). Lower Panel. Sequence logo for proton transfer pathway and active site for clade B CODH and clade F CODH with *Rf*CODH and *Ch*CODH‐II numbering, respectively. For detailed view of the putative proton transfer pathway of *Ch*CODH‐II see Figure S12.

Equally striking are the differences in the proton transfer pathway. In clade F CODHs, a chain of histidine residues provides a proton relay from the protein surface to the active site, essential for coupling CO oxidation to proton release [3]. In *Rf*CODH, this pathway appears altered. First, the aforementioned additional surface loop L1 close to the surface of *Rf*CODH harbours three histidine residues and represents a putative new entry point (Figure 6). Another loop (L3), present in *Ch*CODH‐II but extended in *Rf*CODH, occludes the entry point known for *Ch*CODH‐II. Second, sequence analysis reveals that clade B CODHs contain two conserved substitutions (Glu177, Leu180 in *Rf*CODH) at the terminal positions of the clade F histidine chain (His99, His102, *Ch*CODH‐II numbering, Figure 6), that are described by Kim et al. to be non‐essential but modulate CO oxidation activity greatly [3]. Thirdly, another amino acid mentioned by Kim et al. to be responsible for activity attenuation (Asn262, *Ch*CODH‐II numbering) is consistently exchanged to Ser or Leu in clade B CODHs (Leu341 in *Rf*CODH). These structural variations of the proton transfer pathway in *Rf*CODH and all clade B CODHs are expected to alter proton transfer efficiency. It is conceivable that the three‐histidine loop could adopt alternative conformations under specific conditions (pH, binding of metal ions, temperature, …), enhancing or restricting proton transfer. However, our structure provides no evidence for such flexibility.

The D‐cluster, typically serving as the gateway for electron transfer, shows canonical [4Fe‐4S] architecture with well‐resolved density. Similarly, the B‐cluster, which serves as an electron relay between the D and the C cluster, exhibits the expected [4Fe‐4S] cubane structure, as supported by our EPR spectroscopy. However, the C‐cluster region, despite reasonable density, shows features that may explain the unusual EPR signals we observe. As mentioned earlier, in canonical CODH the [Ni3Fe4S] cluster is coordinated by four cysteines. In clade B CODH this coordination is altered to exchange one cysteine to a carboxylic acid, most often an aspartate (Asp415 in *Rf*CODH). We believe that it is this exchange that alters the C cluster due to a different and more labile coordination.

The structural data provide a compelling rationale for *Rf*CODH's three‐ to five orders of magnitude lower CO oxidation activity compared to canonical CODHs. The enzyme appears to be a monodirectional catalyst, as evidenced by our inability to detect CO_2_ reduction activity and the structural barriers. Whether CO oxidation represents a vestigial activity or whether *Rf*CODH processes CO under specific physiological conditions that overcome these structural constraints remains an open question. The possibility that clade B CODHs utilise entirely different substrates, delivered through the ABC transporter system, represents an attractive alternative hypothesis that warrants further investigation.

### 2.5 Co‐evolution Analysis of Clade B CODH Operons

**FIGURE 7:**
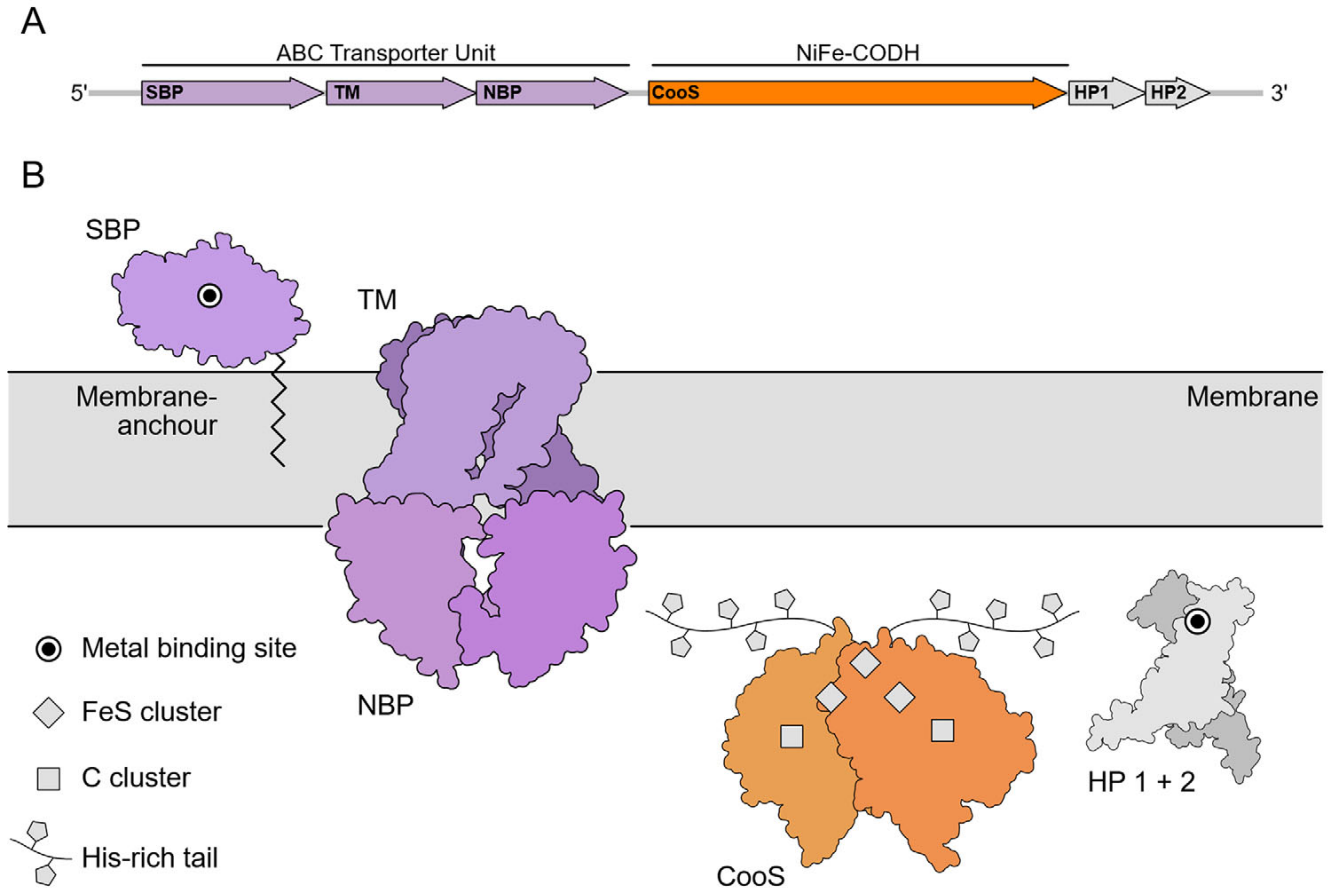
Genetic and structural organization of *Rf*CODH. (A) Operon structure of *Rf*CODH encompassing the canonical clade B architecture: the CODH gene (orange, CooS) flanked by ABC transporter units (purple, substrate binding protein (SBP), nucleotide binding protein (NBP) and transmembrane proteins (TM)) and two conserved hypothetical proteins (grey, HP1 and HP2). (B) Schematic view of operon‐encoded proteins. The SBP is connected to the membrane via a lipid anchor. TM and NBP build a complex in the membrane, with NBP on the cytosolic side. Colour schemes same as in (A).

Our previous work and that of others showed clearly that clade B CODH co‐occur to a high probability with ABC transporter units [12, 13]. So is the case for *Rf*CODH and is illustrated in Figure 7A. Its operon consists of three ABC transporter units: substrate binding protein (SBP), transmembrane protein (TM) and a nucleotide binding protein (NBP). Additionally, two hypothetical, but conserved, proteins (HP1 and HP2) are encoded in proximity to *Rf*CODH (CooS). We aim to learn more about the native chemistry of clade B CODH by understanding the connection with their neighbouring genes.

We used the mirror tree method by Pazos and Valencia [38] to understand to what degree clade B CODH and ABC transporter units are co‐evolved. For this, we generated phylogenetic trees for all ABC transporter subunits and clade B CODH. We extracted the distance matrices and vectorised these matrices to calculate Pearson correlation between clade B CODH and their associated ABC transporter units (Figures S13 and S14), to estimate their co‐evolution.

To control for phylogenetic background signal, we compared CODHs with a phylogenetic marker such as the 16S ribosomal subunit (16S rRNA) and RNA polymerase beta‐subunit (rpoB) from the organisms encoding clade B CODH. Specific co‐evolution should exceed background phylogenetic similarity between CODH and those markers.

The highest correlation was determined between the ABC transporter subunits (0.81 SBP/NBD, 0.87 TM/NBD, 0.91 SBP/TM), all above the threshold of 0.8 for true protein–protein interaction as established by Goh et al. [39] and Pazos et al. [38] (Figure S13). Moderate, though below‐threshold, correlations are observed between CODH and ABC transporter subunits (0.49 CODH/SBP, 0.31 CODH/TM, 0.24 CODH/NBP). These correlations are stronger than those observed between CODH and phylogenetic background markers such as 16S rRNA or rpoB (0.18 CODH/16s RNA, 0.14 CODH/rpoB).

The evolution of clade B CODH correlates best with the evolution of SBP subunits from their respective operons. We propose that this partly results from both proteins interacting with a shared substrate molecule. The below‐threshold correlation (0.49 < 0.80) is explained by their differing cellular localizations: CODHs are likely cytoplasmic, whereas SBPs are located outside the cell (Figure 7B). Consequently, we expected no physical protein–protein interaction. We interpret the observed trend as an indication that both ABC transporter unit and CODH interact with the same molecule, but not with each other. Therefore, either the product or the substrate of CODH is transported by the ABC transporter unit and since ABC transporters with SBPs typically function as importers [40, 41], we infer that the substrate of CODH is being imported via this transporter system. Given its low activity toward CO and the fact that CO can diffuse freely across membranes, we conclude that CO is unlikely to be the primary substrate of clade B CODHs.

The substrate transported by these ABC transporter systems remains unclear. While nickel transport was initially proposed [13], clade B CODH associated SBPs (around 300 amino acids) are significantly smaller than known ABC transporter nickel‐binding proteins (around 500 amino acids) [42]. Additionally, clade B CODH associated SBPs show great phylogenetic similarity to SBPs that bind small anions such as bicarbonate and nitrate [40]. They most closely resemble a zinc‐binding protein from *Staphylococcus aureus* (PDB ID: 3UN6) of unknown function [43], which enabled good structural modelling, but this did not help substrate prediction. Examination of the AlphaFold 3 model of the *Rf*CODH‐associated SBP suggests that it binds a zinc or another metal ion, coordinated by two cysteines and one or two histidines, resembling a zinc‐finger motif (Figure S15). The metal binding motif similarity between SBP and bacterial β‐carbonic anhydrases was also noted; however, structural alignments of known β‐carbonic anhydrase structures (PDB ID: 1EKJ) to the AlphaFold model yielded poor alignments. In literature, we found close relatives to *S. aureus* SBP homologous to clade B CODH associated SBPs, that are known to bind bicarbonate and nitrate (phylogenetic cluster F‐IV as described by Scheepers et al.) [40]. We tested *Rf*CODH towards the reduction of both of these ions and could not see any turnover (Figure 1C). Interestingly, our mirror tree analysis revealed that many clade B CODHs are encoded adjacent to multiple ABC transporter units (Figure S14). These cases were excluded from the primary correlation analysis due to their added genomic complexity but might help triangulate the true substrate of clade B CODHs in future studies.

## 3 Conclusion

Our structural and biochemical characterisation of *Rf*CODH supports that clade B CODHs represent a distinct evolutionary lineage that has diverged from canonical CO‐oxidising enzymes. While maintaining the core CODH scaffold, *Rf*CODH exhibits systematic modifications to substrate access pathways, including occluded gas channels and an altered proton transfer pathway, that fundamentally change its catalytic activity. The reduction in CO oxidation activity, combined with the absence of measurable CO_2_ reduction capability using a standard solution‐based CO‐evolution assay, demonstrates that despite its name and structural homology, *Rf*CODH does not function as a traditional carbon monoxide dehydrogenase. We cannot, however, provide definite proof that there is no CO_2_ reduction activity in *Rf*CODH as this in many characterized CODHs is several orders of magnitude lower than their activity towards CO oxidation. In the case of *Rf*CODH, this would result in activity levels below the detection limit of the assay used in this study. Future studies using more sensitive methods such as protein film electrochemistry could possibly detect a very low activity towards CO_2_ reduction.

The co‐evolution of clade B CODHs with ABC transporter substrate‐binding proteins points toward an alternative physiological role where substrate delivery is mediated through active transport rather than passive gas diffusion.

Our EPR analysis reveals unprecedented oxidised C‐cluster states not previously characterised in CODHs, suggesting that the active site electronic structure has been modified. The additional presence of a confined nickel species and the unique rhombic signals upon thionine oxidation indicate either incomplete cluster assembly in the heterologous host or, more intriguingly, an intrinsically different C‐cluster architecture optimised for alternative chemistry. The weak but detectable CN^−^ binding observed by FTIR spectroscopy confirms the presence of an open coordination site at nickel, preserving the fundamental capacity for substrate coordination while potentially altering the energetics of catalysis.

While our heterologous expression system has enabled the first structural and spectroscopic characterisation of a clade B CODH, the sub‐stoichiometric nickel incorporation (0.2–0.5 Ni/monomer) indicates incomplete maturation, which undoubtedly contributes to the low observed activities. However, this does not single‐handedly explain the three‐ to five orders of magnitude lower CO oxidation activity compared to other canonical CODHs and we also attribute this low activity to altered C‐cluster coordination as well as severely impaired proton‐ and gas transfer.

The novel anaerobic grid preparation approach described here, enables preparation of grids suitable for cryo‐EM studies of oxygen sensitive metalloproteins requiring minimum modifications to existing anaerobic workflows.

Future studies employing native expression systems or reconstitution with appropriate, but yet unknown, maturation factors will be essential to fully realise the catalytic potential and the true natural function of these enzymes. Nevertheless, the present work establishes that clade B CODHs could represent functionally distinct enzymes whose physiological roles extend beyond CO metabolism.

## Author Contributions

**Maximilian Böhm**: conceptualization, writing – original draft, writing – review and editing, investigation. **Vivek Srinivas**: methodology, investigation, writing – review and editing, formal analysis. **Benjamin Wiseman**: investigation, formal analysis. **Ping Huang**: investigation, formal analysis. **Moritz Senger**: writing – review and editing, investigation, formal analysis, funding acquisition. **Martin Högbom**: writing – review and editing, supervision, funding acquisition. **Henrik Land**: conceptualization, writing – original draft, writing – review and editing, supervision, project administration, funding acquisition.

## Conflicts of Interest

The authors declare no conflicts of interest.

## Supporting information



The sequences used in the bioinformatic analysis and the complete sequence of *Rf*CODH cloned in pET11a are supplied as additional files. Tables S1–S2 and Figures S1–S15 can also be found in the electronic supporting information. The authors have cited additional references within the Supporting Information [44–77].**Supporting File 1**: anie73701‐sup‐0001‐SuppMat.docx.

**Supporting File 2**: anie73701‐sup‐0002‐Data.zip.

**Supporting File 3**: anie73701‐sup‐0003‐Data.zip.

## Data Availability

The data that supports the findings of this study are available in the supplementary material of this article. The atomic coordinates of *Rf*CODH and cryo‐EM volume map have been deposited in the Protein Data Bank (PDB) and Electron Microscopy Data Bank (EMDB) under the accession codes **28LW** (pdb_000028lw) and **56604**, respectively.
