# Supplementary material for: Beyond Canonical CO Oxidation: Structural and Evolutionary Insights Into a Non‐Canonical Carbon Monoxide Dehydrogenase
Source: Angew Chem Int Ed Engl. 2026 Jul 24;65(39):e1702233. doi: 10.1002/anie.1702233 (PMC13592312; doi:10.1002/anie.1702233)
Supplement: Supplementary file 1 [file ANIE-65-e1702233-s001.docx]

**Supporting Information**

**Beyond Canonical CO Oxidation: Structural and Evolutionary Insights into a Non-Canonical Carbon Monoxide Dehydrogenase**

Maximilian Böhm^[a]^, Vivek Srinivas^[b]^, Benjamin Wiseman^[b]^, Ping Huang^[a]^, Moritz Senger^[c]^, Martin Högbom^[b]^, and Henrik Land^[a],*^

*Corresponding Author: [henrik.land@kemi.uu.se](mailto:henrik.land@kemi.uu.se)

[a]	Maximilian Böhm, Dr. Ping Huang, Dr. Henrik Land
Department of Chemistry – Ångström Laboratory
Uppsala University
Regementsvägen 10, 752 37 Uppsala, Sweden
E-mail: henrik.land@kemi.uu.se

[b]	Dr. Vivek Srinivas, Prof. Dr. Martin Högbom
Department of Biochemistry and Biophysics and Science for Life Laboratory
Stockholm University
Svante Arrhenius väg 16, 106 91 Stockholm, Sweden

[c]	Dr. Moritz Senger
Department of Chemistry for Life Sciences
Uppsala University
Husargatan 3, 752 37 Uppsala, Sweden

Materials and Methods

Protein Production

The gene encoding *Rf*CODH from *Ruminococcus flavefaciens* ATCC 19208 (WP_028516813.1) was synthesized and cloned in a pET-11a(+) plasmid with a C-terminal StrepII-tag^[1]^ by Genscript® using restriction sites NdeI and BamHI following codon harmonization for expression in *E. coli*. The construct was transformed into *E. coli* BL21 (DE3) ∆*iscR*^[2]^. The cells were grown aerobically at 37 °C, 150 rpm, in a 3 L Erlenmeyer flask in modified LB-medium (20 g/L LB Broth (Lennox) (Sigma-Aldrich L3022), 17 mM KH_2_PO_4_ (Scharlau PO02601000), 72 mM K_2_HPO_4_ (VWR 26932.29), 4 mM L-cysteine (Sigma-Aldrich 168149), 1 g/L ferric ammonium citrate (VWR 0846), 0.5 mM NiCl_2_ (Aldrich 654507), 0.5 % w/v D-glucose (Sigma G8270), 100 µg/mL sodium ampicillin (Sigma-Aldrich A9518)) to an optical density of 0.4 at 600 nm. The cultures were then transferred to 1 L Erlenmeyer flasks, Antifoam C was added (50 µL of 0.1 % v/v, Sigma-Aldrich A8011), the flasks were sealed with butyl rubber septa and purged with nitrogen gas for 30 min. After that sodium fumarate (0.5 % w/v, Sigma-Aldrich F1506), and isopropyl β-D-1-thiogalactopyranoside (0.5 mM, VWR 437145X) was added. The cultures were incubated for 20 h at 20 °C and 150 rpm. The flasks were transferred into an anaerobic wet glovebox (MBraun, O_2_ levels < 5 ppm). Cells were anaerobically harvested through centrifugation (3500 rcf, 15 min), washed with buffer (50 mM Tris (VWR 0497), 20 mM NaCl (Fisher Chemicals S/3120/60), 5 % w/v glycerol (Sigma-Aldrich G7757), adjusted with 10 % HCl (Sigma-Aldrich 258148) to pH 8), and chemically lysed. Lysis was performed by resuspending the cell pellets in lysis buffer (50 mM Tris-HCl, 20 mM NaCl, 5 % w/v glycerol, pH 8, 2 mg/mL lysozyme (Sigma-Aldrich 62971), 0.1 mg/mL DNAse (Sigma-Aldrich DN25), 0.1 mg/mL RNAse (Sigma-Aldrich R5503), 0.1 % sodium deoxycholate (Sigma-Aldrich D6750), 1.0 % saccharose (Sigma-Aldrich 84097), EDTA free protease inhibitor (Roche 05892791001), 2 mM dithiothreitol (DTT, VWR Life Science M109) and letting it rest at room temperature for 1.5 h. The lysate was then ultra-centrifuged at 200 000 xg for 40 min. The soluble fraction was loaded on a 5 mL StrepTrap-XT column (Cytiva 29401323), washed with buffer (50 mM Tris-HCl, 20 mM NaCl, pH 8)) and eluted with elution buffer (50 mM Tris-HCl, 20 mM NaCl, 50 mM biotin (BLD pharm BD21635), 5 % w/v glycerol, 1 mM dithiothreitol, pH 8). The elution fraction was concentrated using 100 kDa molecular cut-off filter Amicon Ultra (Millipore UFC5100). The final protein samples were stored at 4 °C inside the glovebox. Protein concentration was determined using Pierce™ Bradford Plus Protein Assay Kit (thermo scientific 1856209). Metal composition was determined using ICP-OES (Perkin Elmer, Avio 200). SDS-PAGE (Mini-PROTEAN TGX precast Gels, any kD, BioRAD 4569036) was run at 185 V for 45 min, and stained with Readyblue protein gel stain (Sigma-Aldrich RSB).

*Ch*CODH-II (WP_011343033.1) was produced as previously described^[3]^.

Activity Assays

All experiments were performed in an anaerobic glovebox (MBraun, O_2_ levels < 5 ppm) with an argon atmosphere. Buffers and consumables were introduced to the glovebox at least three days before start of the experiment, to ensure that most O_2_ has disrobed. All measurements have been performed in triplicates.

*CO oxidation assay.*

The initial protocol was taken from Benvenuti et al.^[4]^ and adjusted slightly. Protein was diluted with buffer (50 mM buffer, 20 mM NaCl, varying pH) or CO-saturated buffer, to a working concentration of 8 µM, at least 10 min before reaction initiation. In a gastight quartz cuvette, protein (50 µL) was mixed with methyl viologen (MV, Sigma-Aldrich 856177) (50 µL, 50 mM, final concentration 2.5 mM) followed by titration of small amount of sodium dithionite (NaDT, 1-2 µL, 10 mM , Sigma 71699) to a final initial absorbance of 0.2 at 604 nm. The reaction was started by adding CO-saturated buffer up to 1000 µL and the absorbance increase at 604 nm was followed. For pH dependencies MOPS (thermo scientific J19256.A1), TAPS (Sigma-Aldrich T5130) and CHES (Sigma-Aldrich C2885) buffers were used, and pH-adjusted with 2 M NaOH (VWR life science 0583).

*CO_2_ reduction assay.*

The initial protocol was taken from Benvenuti et al.^[4]^ and adjusted. Protein was diluted with buffer (50 mM Tris-HCl, 20 mM NaCl, pH 8), or CO-saturated buffer, to a working concentration of 50 µM. In a gastight quartz cuvette MV (10 µL, 25 mM), NaDT (10 µL, 100 mM), haemoglobin (Sigma H2500, 50 µL, 2 mg/mL) and NaHCO_3_ (Sigma Life S5761, 100 µL, 100 mM) and buffer was mixed to a final volume of 1000 µL. The reaction was started by adding protein (10 µL) and inverting the cuvette. Reaction was followed by tracing absorbance at 429 nm and 419 nm. Alternatively, dmDQ-H (3,11-dimethyl-7,8-dihydro-6*H*-dipyrido[1,2-*a*:2′,1′-*c*][1,4]diazepine-5,9-diium, 10 µL, 25 mM) and EuEGTA (40µL, 50 mM) were used instead of MV and NaDT, respectively. EuEGTA was prepared by mixing ethylene glycol-bis(β-aminoethyl ether)-N,N,N′,N′-tetraacetic acid dissolved in 50 mM NaOH with europium chloride dissolved in water.

*MV oxidation assay.*

The initial protocol was taken from Jeoung et al.^[5]^ and adjusted slightly. In a quartz cuvette, MV (10µL, 1 M) was titrated with a small amount of NaDT (1-2 µL, 10 mM) and buffer (50 mM TAPS-NaOH, 20 mM NaCl, pH 8), to an initial absorbance of 0.7 at 604 nm at 900 µL final volume. Substrate was added (100 µL of either 1 M NaHCO_3_, NaNO_3_ (Merck 1.06537.1000), or NH_2_OH (Sigma-Aldrich 159417)) and a blank was recorded for 2 min to ensure stability of the solution. Protein (1 µL of 400 µM) was added to the solution which was well mixed. The absorbance decrease at 604 nm was followed.

Electron Paramagnetic Resonance (EPR) Spectroscopy

Six EPR samples of *Rf*CODH were prepared, all of which had a final protein concentration of 100 µM protein and a buffer concentration of 50 mM Tris-HCl, 20 mM NaCl, pH 8. The protein was further incubated with either no additional chemical (“as prepared”), 1 mM dithiothreitol (“DTT reduced”), 1 mM NaDT (“NaDT reduced”), CO-saturated buffer (“CO reduced”), 10 mM NaHCO_3_ (“CO_2_ exposed”), or 1 mM thionine (“oxidised”), for 30 min. Samples were the flash frozen in liquid N_2_ and stored in liquid N_2_ until measurement. All spectra were collected using a Bruker ELEXYS E500 spectrometer equipped with a SuperX EPR049 microwave bridge and a cylindrical TE011 ER 4122SHQE cavity, coupled to an Oxford Instruments continuous-flow cryostat. Sample temperatures were controlled via liquid-helium flow regulated by an Oxford Instruments ITC 503 temperature controller. Data acquisition and processing were performed with the Bruker Xepr software package^[6]^, while spectral simulations and fittings were carried out using EasySpin^[7]^ version 6.0.10.

Cyanide binding and Fourier-Transform Infrared (FTIR) spectroscopy

To generate cyanide(CN^-^) bound CODHs the protocol of Ciaccafava et al.^[8]^ was followed. An aliquot of 6 µL enzyme (400 µM) was added to 500 µL buffer (Tris–HCl 50 mM pH 8, NaCl 100 mM, NaDT 4 mM) and incubated for 45 min at room temperature. Potassium cyanide (5 µL of 100 mM) was added, followed by 30 min incubation with occasional gentle inversion of the tube. The reaction mixture was then loaded on a 100 kDa molecular cutoff filter Amicon Ultra (Millipore UFC5100), washed with buffer, and ultimately concentrated to a final volume of 10 µL. From this, 3 µL (of approx. 240 µM) was placed on an Attenuated total reflection (ATR) crystal in an anoxic glovebox. The ATR unit (BioRadII, Harrick) was gas tight sealed with a custom-built PEEK cell and mounted in the FTIR spectrometer (Vertex V70v, Bruker)^[9]^. Samples were then dried under 100% nitrogen. Spectra were collected at a resolution of 2 cm⁻¹ with a scanner velocity of 80 kHz and averaged over a variable number of scans (typically 100). All measurements were carried out under ambient pressure and temperature.

Anaerobic cryo-EM grid preparation

Grid preparation was performed in an MBraun anaerobic glovebox, maintained at 1–2 ppm O₂, under a nitrogen atmosphere. Quantifoil R1.2/1.3 Cu 300 mesh holey carbon grids (Quantifoil Micro Tools) were glow discharged (20 mA, 40 s) using a PELCO easiGlow glow discharge cleaning system (Ted Pella, Inc.) prior to transfer into the glovebox. Ethane was solidified as described previously^[10]^. Briefly, once liquid ethane was prepared, the ethane cup was transferred into a liquid nitrogen dewar and submerged halfway, after a few minutes and complete solidification it was fully submerged. The glovebox deep well was precooled to cryogenic temperature using a liquid nitrogen dewar prior to transfer of the cup. The solidified ethane was transferred through the glovebox antechamber and moved into the deep well using the stage, where it was allowed to liquefy. A grid box was then placed at the bottom of the ethane cup for grid storage. A 5 μL aliquot of *Rf*CODH at 1 mg/mL was applied to a glow-discharged grid held with cross-locking tweezers, incubated for 10 s, manually blotted, followed by immediate plunge freezing into ethane and placed into the submerged grid box. The stage holding the ethane cup was moved out of the cryogenic deep well only briefly during vitrification and the grids are kept continuously submerged in liquid ethane. After all the grids were prepared, the ethane cup was removed from the glovebox and immediately transferred into liquid nitrogen. The grids were clipped and stored in liquid nitrogen until screening. This protocol minimizes the cryogen introduced into the glovebox only to the small ethane cup volume while maintaining the liquid nitrogen temperatures using an external reservoir. As a result, grid preparation is performed with limited gaseous expansion inside the glovebox, avoiding dangerous rapid pressure build-up due to any intentional or unintentional cryogen boil off. The glovebox grid preparation setup used is shown in Fig S5.

Cryo-EM data collection and structure determination

The grids were screened on a Talos Arctica microscope equipped with Falcon 4i detector (Thermo Fisher Scientific). Data was collected from a selected grid on a Titan Krios G2 microscope (T) operated at 300 kV, equipped with a Gatan K3 BioQuantum, at the Swedish National Cryo-EM Facility. A total of 16,036 micrographs were collected in electron counting mode at a magnification of 130000x corresponding to 0.65 Å/pix with a total exposure of 41.65 e⁻/Å² over 39 frames. CryoSPARC^[11]^ was used to perform patch motion correction, patch CTF estimation, particle picking using Topaz^[12]^, 2D classification, and non-uniform refinement. The data processing workflow is outlined in Fig. Y. An AlphaFold^[13]^ predicted model of RfCODH was docked into the resulting map using ChimeraX^[14]^ to generate an initial model. Model building and iterative adjustment were performed in COOT^[15]^ and ISOLDE^[16]^. The structure was refined over multiple rounds of Real-space refinement in Phenix^[17]^.

Bioinformatics

*Tree building*

Accession numbers for clade B and F CODHs were taken from our previous work^[18]^. Sequence data was retrieved from the NCBI nr protein database using Entrez v23.5^[19]^, yielding 315 and 249 sequences, respectively. Associated neighbours for clade B CODH were identified using utilities presented in previous work, with 300 bp intergenic space, 50 bp overlap between genes and a maximum of 8 neighbours upstream and downstream of clade B CODH, using our custom script.^[18,20,21]^ From this, sequences that were annotated as “ABC transporter substrate-binding protein”, “permease” or “ATP-binding protein” were collected. Permease relates to the transmembrane proteins in the ABC transporter units. ATP-binding protein relates to the nucleotide binding proteins. We gathered 199, 210, and 198 sequences respectively. Alignments were performed using MAFFT’s L-INS-I v7.526 algorithm.^[22]^ Alignments for tree building were trimmed using trimAl v1.4.rev22 *automated1*^[23]^. Trees were generated using IQ-TREE v2.0.7^[24]^ using LG+G4 as model, determined to be best suited for clade B CODH dataset by IQ-TREE’s model finder^[25]^ and used throughout for consistency. Ultrafast bootstrapping SH-aLRT were performed with 1000 replicates. The ABC-SBP tree was further refined by removing sequences with too long branches (18) to ensure an undistorted tree. The sequences were then realigned, trimmed and a new tree was generated as described above. All sequences are available in the supporting files as *.fasta* files. Sequence logos were generated with R packages ggseqlogo v0.2.2^[26]^ Biostrings v3.22^[27]^ and msa v1.42.0^[28]^.

*Correlation calculation*

As negative control for mirror tree correlation the RNA sequences of the 16S ribosomal subunit (16S) and the protein sequences from the β subunit of bacterial RNA polymerase (rpoB) were retrieved from NCBI nr protein database using a custom R script^[29]^ using Biostrings v3.22^[27]^, rentrez v1.2.3^[30]^, and stringr^[31]^. All sequences are available in the supporting files as *.fasta* files. Duplicates were removed and the sequences were aligned and trimmed as stated above. Initial trees were constructed, with TIMe3+I+G4 and the LG+G4 model for 16S and rpoB, respectively. Long branches were removed from the initial sequence batches and the process of tree building was once more repeated. The trees were used to generate distance matrices using the ape v5.8-1 package.^[32]^. The matrices were flattened and Pearson correlation between all available trees was calculated. For data processing and plotting R v4.5.1^[33]^ and tidyverse^[34]^ was used. For fast and easy handling of sequence data, seqkit v2.9.0^[35]^ was used. To visualise the mirror trees, tanglegrams were generated using the phytools v2.5-2^[36]^ package.

*Gas channel prediction*

The gas channel was predicted from the cryo-EM structure using the Caver 3.0.3^[37]^ pymol v3.1.1^[37,38]^ plugin. To select the appropriate probe size, a CODH crystal structure obtained under xenon (PDB ID: 7TSJ^[39]^) was used and Caver was run with different sphere sizes (0.6 to 1.4 Å) to replicate the experimentally observed gas channels. Ultimately, a sphere radius of 1.1 Å was chosen.

*AlphaFold modelling*

AlphaFold^[13]^ models were generated for all proteins of the *Rf*CODH encoding operon. The ABC transporter module was modelled as two ATP-binding proteins (WP_028516814.1), two permeases (WP_028516815.1) and two ADP ligands. The ABC transporter substrate-binding protein (WP_028516816.1) was modelled as monomer with a zinc ion. *Rf*CODH (WP_028516813.1) was modelled as homodimer before we obtained the cryo-EM structure. The two hypothetical proteins (WP_028516812.1, WP_028516811.1) downstream from *Rf*CODH were modelled with different ratios (Fig. S1), and it was found that a heterodimer gave the best confidence values. This choice was further supported by highly conserved residues at the proteins’ interfaces.

Tables

Table S1. **Overview of different EPR species**.

| State | CODH | g_1_ | g_2_ | g_3_ | g_av_ | Reference |
| --- | --- | --- | --- | --- | --- | --- |
| B_red_ | *Rf*CODH | 2.057 | 1.956 | 1.908 | 1.974 | This study |
|  |  | 2.062 | 1.954 | 1.911 | 1.976 | This study |
|  | *Ch*CODH-II | 2.03 | 1.93 | 1.90 | 1.95 | ^[40]^ |
| C_red1_ | *Rf*CODH | 2.030 | 1.897 | 1.705 | 1.877 | This study |
|  | *Ch*CODH-II | 1.97 | 1.82 | 1.64 | 1.81 | ^[40]^ |
| B_red*_ | *Rf*CODH | 2.055 | 1.955 | 1.909 | 1.973 | This study |
|  |  | 2.061 | 1.955 | 1.915 | 1.977 | This study |
| C_alt-red_ | *Rf*CODH | 2.282 | 2.213 | 2.200 | 2.232 | This study |
| C_alt-ox_ | *Rf*CODH | 2.022 | 2.011 | 1.919 | 1.984 | This study |
|  | *Rf*CODH | 2.011 | 1.974 | 1.963 | 1.983 | This study |

Table S2. **Cryo-EM data collection and refinement statistics**

| **Data Collection Processing** | |
| --- | --- |
| Magnification | 130000x |
| Voltage (kV) | 300 |
| Spherical Aberration (mm) | 2.7 |
| Total Dose (e^-^/Å^2^) | 41.65 |
| Defocus range (µm) | -0.6 to -2.0 |
| Pixel size (Å/pix) | 0.65 |
| Total micrographs | 16,036 |
| **CryoSPARC processing** | |
| Total particles count | 3,051,224 |
| Extraction box size (pixels) | 320 |
| Final particles count | 123,492 |
| Map resolution (Å) | 2.53 |
| FSC threshold | 0.143 |
| Symmetry imposed | C1 |
| **Model and refinement statics** | |
| Chains | 2 |
| Nonhydrogen atoms | 10130 |
| Amino acid Residues | 1319 |
| Ligands | SF4: 3 |
| Bonds (RMSD) |  |
| Length (Å) (# > 4σ) | 0.005 (0) |
| Angles (°) (# > 4σ) | 1.047 (0) |
| MolProbity score | 1.70 |
| Clash score | 4.69 |
| Ramachandran plot (%) |  |
| Outliers | 0.08 |
| Allowed | 3.66 |
| Favored | 96.26 |
| Rotamer outliers (%) | 1.86 |
| CaBLAM outliers (%) | 1.30 |
| ADP (B-factors) |  |
| Iso/Aniso (#) | 10130/0 |
| Protein (min/max/mean) | 78.54/181.39/116.33 |
| Ligand (SF4) | 95.02/131.27/110.66 |
| Resolution Estimates (Masked) (Å)  d FSC model (0/0.143/0.5) | 2.5/2.5/2.9 |
| Resolution Estimates (Unmasked) (Å)  d FSC model (0/0.143/0.5) | 2.5/2.5/3.1 |
| Model vs. Data |  |
| CC (Mask/Box/Peaks/Volume/mean-ligands) | 0.80/0.77/0.66/0.80/0.91 |

Figures

Figure S1. **SDS-PAGE of *Rf*CODH purification.** Lane 1: *E. coli* cells before induction; lane 2: *E. coli* cells after induction; lane 3: lysed cells, lane 4: insoluble fraction after ultra-centrifugation; lane 5: soluble fraction after ultra-centrifugation; lane 6: flow through during loading of soluble fraction on affinity column; lane 7 and 8: wash fractions; L: protein page ruler; P1: purified *Rf*CODH; P2: purified *Rf*CODH with 10x higher concentration. The dotted red line shows the section of the SDS-PAGE gel shown in Figure 1.

Figure S2. **EPR spectra of *Rf*CODH**. EPR spectra of *Rf*CODH in different redox states: as prepared, DTT reduced, NaDT reduced, CO reduced, CO₂ exposed, and oxidised. Spectra were recorded at 5 K, 15 G, 100 kHz, 9.28 GHz, and 20 µW.

Figure S3. **Power-Temperature studies for g 2.2 signal in CO reduced *Rf*CODH.** (A) Square root of power was plotted against signals amplitude at different temperature. (B) Excerpt of data from (A) to show linear range for temperatures between 7–20 K. (C) and (D) are two versions of Curie Plots. (C) Inverse Temperature against signal amplitude, a linear correlation would be expected if Curie Law is followed. (D) Temperature against signal amplitude multiplied by temperature, a horizontal line would be expected if Curie Law is followed. All data was collected at an X-band EPR. 15 G, 100 kHz, 9.37 GHz.

Figure S4. **Power-Temperature study on g 1.74 signal of CO reduced *Rf*CODH**. Square root of power against the signal amplitude at varying temperatures. (A) A linear trend was observed for 20 K for the full power range. (B) A linear trend was also observed for lower temperature (5 K and 7 K) at lower power. All data was collected at an X-band EPR. 15 G, 100 kHz, 9.37 GHz.

Figure S5. **Anaerobic glovebox setup for cryo-EM grid preparation.** The glovebox grid preparation workflow follows the anaerobic handling procedure described in our previous work^[10]^, with modifications introduced here to adapt for manual blotting for single particle cryo-EM grid preparation. (A) Overview of our glovebox setup highlighting the deep well with the liquid nitrogen in the raised position. (B) Close up picture of the deep well when the liquid nitrogen dewar is lowered. (C) Picture of the deep well as seen inside the glovebox. (D) Working surface of the glovebox showing, (i) the stage designed to hold the ethane cup to facilitate transfer in and out of the deep well, (ii) glow-discharged grids, (iii) cross-locking tweezers, and (iv) blotting paper used during manual plunge freezing.

Figure S6. **Cryo-EM micrograph processing workflow outline of *Rf*CODH in cryoSPARC**. A total of 16,036 micrographs were collected and motion corrected followed by CTF estimation. Particles were initially manually picked to train a Topaz model and then Topaz extract was used to pick total of 3,051,224 particles. All particles were extracted with a box size of 320 pixels. After 2D classification 1,976,790 particles were selected for *ab-initio* reconstruction. The best class was subjected successive rounds of *ab-initio* reconstruction with maximum resolution of 4 Å. The resulting best class with 123,492 particles were selected for non-uniform refinement with C1 symmetry. The final reconstructed coulomb potential volume reached a resolution of 2.53 Å (FSC 0.143). The NU-refined map was further processed with deepEMhancer and used only to generate figures.

Figure S7. **Active site comparison between *Rf*CODH and *Ch*CODH-II.** (A) Sequence logos of selected residues from cluster regions of clade B and clade F CODH, with *Rf*CODH and *Ch*CODH-II numbering, respectively. (B) Structural alignment of *Ch*CODH-II (pink, PDB ID: 4UDX) and *Rf*CODH (grey) with *Rf*CODH numbering. Residues that differ between the two are highlighted in bold.

Figure S8. **Resolution distribution of the final *Rf*CODH volume.** Three orthogonal views of the map are shown with high resolution regions in blue to lower resolution regions in red. The colour scale indicates resolution in Å.

Figure S9. **Ligand binding sites in *Rf*CODH.** Local cryo-EM volume of metal cofactor sites in *Rf*CODH. (A, C) B cluster map volumes from chain A and B respectively, (B) D cluster map volume, (D, E) C cluster map volumes from chain A and B respectively. The B and D cluster regions showed well-defined volumes enabling confident modelling of [4Fe4S] (SF4) ligands. However, the C cluster region showed heterogeneous and irregular coulomb volume that could not be reliably modelled without misinterpretation. A deepEMhancer map was used to generate figures.

Figure S10. **Gas Channel of *Rf*CODH.** The gas channel of *Rf*CODH was predicted with CAVER 3.0^[37]^ with a probe radius of 0.9 Å. In gray *Rf*CODH, in light blue *Nv*CODH (PDB ID: 7TSJ), in petrol Xenon atoms from *Nv*CODH structure indicating its gas channels. Orange highlights residues of *Rf*CODH that are in conflict with the gas channel observed in *Nv*CODH.

Figure S11. **Multiple sequence alignment of selected CODHs**. Positions discussed by Biester *et al.* (2022) are framed and coloured with the Clustal colours scheme. Positions to be important for proton transfer in canonical CODH as suggested by Kim *et al.* (2004) are marked with a +, positions believed to be important for substrate transport as suggested by Biester *et al.* (2022) are coloured and framed, positions in *Rf*CODH that are in conflict with this predicted gas channel(s) are marked with an *. Alignment was performed using MAFFT’s L-INS-I v7.526 (Katoh et al. 2005)^[41]^. For presentation JalView v2.11.5.1^[42]^ was used.

Figure S12. **Proton transfer pathway of *Ch*CODH-II.** The proton transfer pathway of *Ch*CODH-II as suggested by Kim et al.^[43]^ Pink structure is based on the PDB ID 4UDX. *Ch*CODH-II amino acid numbering.

Figure S13. **Mirror tree comparisons.** (A) Scatter plot of mirror tree Pearson correlations. Each point represents the correlation between phylogenetic distance matrices from pairs of trees (Figure S13). The y-axis indicates the tree pairs compared, while the x-axis shows their Pearson correlation coefficient (B). (B) Flattened distance matrices from different tree pairs were compared, and Pearson correlation coefficients were calculated. Each panel shows the relationship between paired distances; the red line represents a linear fit through the data. Positive controls consist of comparison between ABC transporter subunits (substrate-binding protein (SBP), permease (TM), ATP-binding protein (NBP)), and negative controls are represented by correlation between CODH and host organism’s 16S rRNA and rpoB trees. Trees were constructed as described: sequences were retrieved, aligned (MAFFT L-INS-i^[22,41]^), trimmed (trimAl^[23]^), and phylogenies inferred with iq-tree2^[24]^ using LG+G4 or TIMe3+I+G4, followed by manual pruning. Correlations were calculated from flattened distance matrices generated with the ape package in R.

Figure S14. **Tanglegrams of mirror tree comparisons.** Phylogenetic trees were constructed as described above. Tanglegrams visualise the correspondence between CODH and its neighbours: (A) CODH vs ABC substrate-binding protein (SBP), (B) CODH vs permeases, and (C) CODH vs ATP-binding proteins. Lines connect orthologous sequences across paired trees, illustrating topological similarity consistent with correlated evolution. Line colour indicates connections per CODH, one connection grey, two connections red, three and more connections blue.

Figure S15. **AlphaFold prediction of *Rf*CODH associated SBP**. AlphaFold 3^[13]^ predicted structure was modeled as a monomer with an additional zinc atom. N terminal tail that is part of the signaling sequence for later lipofication is not shown (Continued Tail). Residues are coloured by degree of conversion based on sequence alignment used for SBP tree generation, see above, blue non conserved, red highly conserved. Highly conserved residues (>95% conservation) are shown as sticks.

References

[1]	T. G. Schmidt, A. Skerra, “The Strep-tag system for one-step purification and high-affinity detection or capturing of proteins” *Nat Protoc* **2007**, *2*, 1528–1535.

[2]	M. K. Akhtar, P. R. Jones, “Deletion of iscR stimulates recombinant clostridial Fe–Fe hydrogenase activity and H2-accumulation in Escherichia coli BL21(DE3)” *Appl Microbiol Biotechnol* **2008**, *78*, 853–862.

[3]	M. V. Pavliuk, M. Böhm, J. Wilhelmsen, S. Hardt, H. Land, H. Tian, “Photobiocatalytic CO 2 reduction into CO by organic nanorod-carbon monoxide dehydrogenase assemblies: surfactant matters” *Chemical Science* **2024**, *15*, 16789–16795.

[4]	M. Benvenuti, M. Meneghello, C. Guendon, A. Jacq-Bailly, J. H. Jeoung, H. Dobbek, C. Leger, V. Fourmond, S. Dementin, “The two CO-dehydrogenases of Thermococcus sp. AM4” *Biochim Biophys Acta Bioenerg* **2020**, *1861*, 148188.

[5]	J. H. Jeoung, J. Fesseler, L. Domnik, F. Klemke, M. Sinnreich, C. Teutloff, H. Dobbek, “A Morphing [4Fe-3S-nO]-Cluster within a Carbon Monoxide Dehydrogenase Scaffold” *Angew Chem Int Ed Engl* **2022**, *61*, e202117000.

[6]	C. Albers, T. Baecker, H. Barth, S. Corsi, M. Dressel, A. Gibbs, M. Heichel, G. Heindl, D. Hoelzer, F. Jent, H. Meling, P. Printz, J. Schulte, K. Schwab, T.-Q. Ye **2016**.

[7]	S. Stoll, A. Schweiger, “EasySpin, a comprehensive software package for spectral simulation and analysis in EPR” *Journal of Magnetic Resonance* **2006**, *178*, 42–55.

[8]	A. Ciaccafava, D. Tombolelli, L. Domnik, J. Fesseler, J. H. Jeoung, H. Dobbek, M. A. Mroginski, I. Zebger, P. Hildebrandt, “When the inhibitor tells more than the substrate: the cyanide-bound state of a carbon monoxide dehydrogenase” *Chem Sci* **2016**, *7*, 3162–3171.

[9]	M. Senger, T. Kernmayr, M. Lorenzi, H. J. Redman, G. Berggren, “Hydride state accumulation in native [FeFe]-hydrogenase with the physiological reductant H2 supports its catalytic relevance” *Chem. Commun.* **2022**, *58*, 7184–7187.

[10]	J. Xu, V. Srinivas, R. Kumar, L. Pacoste, Y. Guo, T. Yang, C. C. Sun, M. Högbom, X. Zou, H. Xu, “Unveiling the Structure of Anhydrous Sodium Valproate with 3D Electron Diffraction and a Facile Sample Preparation Workflow” *ACS Cent. Sci.* **2025**, *11*, 960–966.

[11]	A. Punjani, J. L. Rubinstein, D. J. Fleet, M. A. Brubaker, “cryoSPARC: algorithms for rapid unsupervised cryo-EM structure determination” *Nat Methods* **2017**, *14*, 290–296.

[12]	T. Bepler, A. Morin, M. Rapp, J. Brasch, L. Shapiro, A. J. Noble, B. Berger, “Positive-unlabeled convolutional neural networks for particle picking in cryo-electron micrographs” *Nat Methods* **2019**, *16*, 1153–1160.

[13]	J. Abramson, J. Adler, J. Dunger, R. Evans, T. Green, A. Pritzel, O. Ronneberger, L. Willmore, A. J. Ballard, J. Bambrick, S. W. Bodenstein, D. A. Evans, C.-C. Hung, M. O’Neill, D. Reiman, K. Tunyasuvunakool, Z. Wu, A. Žemgulytė, E. Arvaniti, C. Beattie, O. Bertolli, A. Bridgland, A. Cherepanov, M. Congreve, A. I. Cowen-Rivers, A. Cowie, M. Figurnov, F. B. Fuchs, H. Gladman, R. Jain, Y. A. Khan, C. M. R. Low, K. Perlin, A. Potapenko, P. Savy, S. Singh, A. Stecula, A. Thillaisundaram, C. Tong, S. Yakneen, E. D. Zhong, M. Zielinski, A. Žídek, V. Bapst, P. Kohli, M. Jaderberg, D. Hassabis, J. M. Jumper, “Accurate structure prediction of biomolecular interactions with AlphaFold 3” *Nature* **2024**, *630*, 493–500.

[14]	E. F. Pettersen, T. D. Goddard, C. C. Huang, E. C. Meng, G. S. Couch, T. I. Croll, J. H. Morris, T. E. Ferrin, “UCSF ChimeraX : Structure visualization for researchers, educators, and developers” *Protein Science* **2021**, *30*, 70–82.

[15]	P. Emsley, K. Cowtan, “*Coot* : model-building tools for molecular graphics” *Acta Crystallogr D Biol Crystallogr* **2004**, *60*, 2126–2132.

[16]	T. I. Croll, “*ISOLDE* : a physically realistic environment for model building into low-resolution electron-density maps” *Acta Crystallogr D Struct Biol* **2018**, *74*, 519–530.

[17]	D. Liebschner, P. V. Afonine, M. L. Baker, G. Bunkóczi, V. B. Chen, T. I. Croll, B. Hintze, L.-W. Hung, S. Jain, A. J. McCoy, N. W. Moriarty, R. D. Oeffner, B. K. Poon, M. G. Prisant, R. J. Read, J. S. Richardson, D. C. Richardson, M. D. Sammito, O. V. Sobolev, D. H. Stockwell, T. C. Terwilliger, A. G. Urzhumtsev, L. L. Videau, C. J. Williams, P. D. Adams, “Macromolecular structure determination using X-rays, neutrons and electrons: recent developments in *Phenix*” *Acta Crystallogr D Struct Biol* **2019**, *75*, 861–877.

[18]	M. Böhm, H. Land, “Investigating the native functions of [NiFe]-carbon monoxide dehydrogenases through genomic context analysis” *eLife* **2026**, *15*, DOI 10.7554/eLife.108780.1.

[19]	E. Sayers in *Entrez Programming Utilities Help [Internet]*, National Center For Biotechnology Information (US), **2022**.

[20]	M. Böhm **2025**, DOI https://doi.org/10.5281/zenodo.16736722.

[21]	M. Böhm **2025**, DOI https://doi.org/10.5281/zenodo.16736767.

[22]	K. Katoh, D. M. Standley, “MAFFT Multiple Sequence Alignment Software Version 7: Improvements in Performance and Usability” *Mol Biol Evol* **2013**, *30*, 772–780.

[23]	S. Capella-Gutiérrez, J. M. Silla-Martínez, T. Gabaldón, “trimAl: a tool for automated alignment trimming in large-scale phylogenetic analyses” *Bioinformatics* **2009**, *25*, 1972–1973.

[24]	B. Q. Minh, H. A. Schmidt, O. Chernomor, D. Schrempf, M. D. Woodhams, A. von Haeseler, R. Lanfear, “IQ-TREE 2: New Models and Efficient Methods for Phylogenetic Inference in the Genomic Era” *Molecular Biology and Evolution* **2020**, *37*, 1530–1534.

[25]	S. Kalyaanamoorthy, B. Q. Minh, T. K. F. Wong, A. von Haeseler, L. S. Jermiin, “ModelFinder: fast model selection for accurate phylogenetic estimates” *Nat Methods* **2017**, *14*, 587–589.

[26]	O. Wagih, “ggseqlogo: a versatile R package for drawing sequence logos” *Bioinformatics* **2017**, *33*, 3645–3647.

[27]	H. Pagès, P. Aboyoun **2017**, DOI 10.18129/B9.BIOC.BIOSTRINGS.

[28]	U. Bodenhofer, E. Bonatesta, C. Horejš-Kainrath, S. Hochreiter, “msa: an R package for multiple sequence alignment” *Bioinformatics* **2015**, *31*, 3997–3999.

[29]	M. Böhm **2026**, DOI https://doi.org/10.5281/zenodo.18506471.

[30]	D. J. Winter, “rentrez: an R package for the NCBI eUtils API” *The R Journal* **2017**, *9*, 520--526.

[31]	H. Wickham **2025**.

[32]	E. Paradis, K. Schliep, “ape 5.0: an environment for modern phylogenetics and evolutionary analyses in R” *Bioinformatics* **2019**, *35*, 526–528.

[33]	R Core Team **2023**.

[34]	H. Wickham, M. Averick, J. Bryan, W. Chang, L. McGowan, R. François, G. Grolemund, A. Hayes, L. Henry, J. Hester, M. Kuhn, T. Pedersen, E. Miller, S. Bache, K. Müller, J. Ooms, D. Robinson, D. Seidel, V. Spinu, K. Takahashi, D. Vaughan, C. Wilke, K. Woo, H. Yutani, “Welcome to the Tidyverse” *JOSS* **2019**, *4*, 1686.

[35]	W. Shen, S. Le, Y. Li, F. Hu, “SeqKit: A Cross-Platform and Ultrafast Toolkit for FASTA/Q File Manipulation” *PLOS ONE* **2016**, *11*, e0163962.

[36]	L. J. Revell, “phytools 2.0: an updated R ecosystem for phylogenetic comparative methods (and other things)” *PeerJ* **2024**, *12*, e16505.

[37]	E. Chovancova, A. Pavelka, P. Benes, O. Strnad, J. Brezovsky, B. Kozlikova, A. Gora, V. Sustr, M. Klvana, P. Medek, L. Biedermannova, J. Sochor, J. Damborsky, “CAVER 3.0: A Tool for the Analysis of Transport Pathways in Dynamic Protein Structures” *PLOS Computational Biology* **2012**, *8*, e1002708.

[38]	Schrödinger, LLC **2024**.

[39]	A. Biester, S. Dementin, C. L. Drennan, “Visualizing the gas channel of a monofunctional carbon monoxide dehydrogenase” *Journal of Inorganic Biochemistry* **2022**, DOI 10.1016/j.jinorgbio.2022.111774.

[40]	Y. Basak, C. Lorent, J.-H. Jeoung, I. Zebger, H. Dobbek, “Metalloradical-driven enzymatic CO2 reduction by a dynamic Ni–Fe cluster” *Nat Catal* **2025**, 1–10.

[41]	K. Katoh, K. Kuma, H. Toh, T. Miyata, “MAFFT version 5: improvement in accuracy of multiple sequence alignment” *Nucleic Acids Res* **2005**, *33*, 511–518.

[42]	A. M. Waterhouse, J. B. Procter, D. M. A. Martin, M. Clamp, G. J. Barton, “Jalview Version 2—a multiple sequence alignment editor and analysis workbench” *Bioinformatics* **2009**, *25*, 1189–1191.

[43]	E. J. Kim, J. Feng, M. R. Bramlett, P. A. Lindahl, “Evidence for a Proton Transfer Network and a Required Persulfide-Bond-Forming Cysteine Residue in Ni-Containing Carbon Monoxide Dehydrogenases” *Biochemistry* **2004**, *43*, 5728–5734.
